# The impact of Bisphenol A on the endophytic bacterial community and transcriptome of soybean seedlings

**DOI:** 10.1016/j.isci.2025.112208

**Published:** 2025-03-13

**Authors:** Ke Wang, Nana Zhong, Manli Yang, Wen Tian, Yaohuan Zhu, Changjiang Huang, Lin Zhao, Xun Liu, Jun Tang, Yuqing Miao, Yuntong Liu, Yu Lei, Chuansheng Wu

**Affiliations:** 1 Anhui Province Key Laboratory of Pollution Damage and Biological Control for Huaihe River Basin, Fuyang Normal University, Fuyang 236037, China; 2 Anhui Provincial Key Laboratory of Earth Surface Processes and Regional Response in the Yangtze-Huaihe River Basin, School of Geography and Tourism, Anhui Normal University, Wuhu 241002, China; 3 School of Chemical Biology and Environment, Yuxi Normal University, Yuxi 653100, China; 4 Department of Ecology, College of Life and Environmental Sciences, Hangzhou Normal University, Hangzhou 311121, China

**Keywords:** Microbiology, Plant biology, Plant ecology, Agricultural science

## Abstract

Bisphenol A (BPA) is widely acknowledged as an endocrine disruptor, and its toxicological effects have garnered considerable research interest. In this investigation, a soil pot experiment was conducted to examine the consequences of sustained BPA exposure on the growth of soybean seedlings, the transcriptome, and the endophytic bacterial community. We observed a substantial inhibition in soybean seedling growth. Transcriptome analysis showed that growth-related genes in both leaves and roots were markedly downregulated following BPA treatment. Intriguingly, BPA considerably increased the abundance and diversity of endophytic bacteria in leaves while suppressing beta diversity in roots. A significant association was identified between amplicon sequence variants and differentially expressed genes under BPA treatment in the leaves and roots. These findings illuminate the effects of continuous exposure to BPA on the transcriptome and endophyte of soybean seedlings, which may collectively impair soybean seedling growth, offering valuable insights into BPA toxicity in plants.

## Highlights

• The biomass of soybean seedlings significantly decreased
• The endophyte community structure was altered
• Growth-related gene expression was inhibited in leaves and roots
• Endophytes regulated the gene expression of resistance to stress

## Introduction

Bisphenol A (BPA), a characteristic endocrine disruptor,^1^ exhibits a propensity for soil accumulation owing to its widespread production and utilization, facilitated by processes such as sewage irrigation, solid waste recycling, and atmospheric sedimentation.^2^^,^^3^^,^^4^^,^^5^^,^^6^ Plants can uptake BPA from the soil, resulting in bioaccumulation and toxicology that adversely affect human health.^1^^,^^7^^,^^8^^,^^9^^,^^10^^,^^11^ Hence, it is imperative to undertake deeper investigations to elucidate the impacts of BPA on plants and the underlying mechanisms governing its actions.

Numerous studies have been conducted to investigate the adverse effects of BPA on plants,^10^ including its impact on biomass,^8^^,^^12^ plant hormone regulation,^13^^,^^14^ mineral element absorption,^15^^,^^16^ root system development,^17^^,^^18^ and photosynthesis.^19^^,^^20^ At the molecular level, microarray analysis has revealed an overrepresentation of genes involved in the detoxification system in *Arabidopsis* in the presence of BPA.^21^ Additionally, Xiang et al.^22^ confirmed the downregulation of photosynthesis-related genes in *Cylindrospermopsis raciborskii*. RNA sequencing (RNA-seq) technology offers comprehensive information about gene expression, which is extensively employed in the investigation of stress response mechanisms in various plant species, such as *Arabidopsis thaliana*,^23^ rice (*Oryza sativa* L.),^24^ and ryegrass.^25^ Therefore, utilizing transcriptome holds to provide insights into the underlying mechanism of BPA-induced phytotoxicity.

Previous research has examined the impact of BPA on microorganisms, with a specific focus on the rhizosphere and sediment.^26^^,^^27^^,^^28^^,^^29^^,^^30^ For instance, when adding BPA, Tong et al.^27^ documented an increased abundance of *Pseudomonas* and *Lutibacter* genera in rhizosphere soils. Similarly, another study revealed a notable increase in *Novosphingobium* and *Croceicoccus* in forest sediment due to BPA biodegradation.^30^ These studies demonstrate that BPA significantly influences microbial community structure and function.^26^^,^^30^ However, limited attention has been devoted to examining the ramifications of BPA on endophytic bacterial communities. A research study reported that the presence of mixed pollution (Zn, Ni, Cd, BPA, SMX, and CIP) at high levels had a detrimental impact on the alpha diversity index of the root endophytic microbial community in *Juncus acutus*.^31^ Consequently, investigation of plant endophyte communities is necessary to understand the consequences of BPA pollution in soil.

The presence of endophytic bacteria has been found to impact the expression of plant genes, leading to increased plant stress tolerance and improved plant growth.^32^^,^^33^^,^^34^^,^^35^^,^^36^ For example, *Bacillus thuringiensis* PM25 has been found to induce overexpression of stress-related genes (APX and SOD) in maize under salt stress, resulting in increased growth.^37^ The endophyte EF0801 has been shown to regulate transcription-related processes, including antioxidant defense and plant hormone synthesis, thereby improving rice growth and development under Pb stress.^38^ While previous research has primarily investigated the association between culturable endophytes and host gene expression,^35^^,^^37^^,^^38^^,^^39^^,^^40^ the impact of the endophyte community on the transcriptome remains relatively unexplored.

Based on the information above, we hypothesized that (1) BPA has the potential to induce changes in both plant endophyte populations and transcriptomes and (2) endophytes play a regulatory role in host gene expression, ultimately leading to the inhibition of plant growth. Soybeans (*Glycine max*), a plant species recommended by the United States Environmental Protection Agency^41^ for evaluating the impact of pollutants, serve as a crucial protein source.^42^ This study employed soybean as a model plant to examine the modifications in physiological indices, transcriptome, and endophyte communities after continuous exposure to BPA. The aims of this investigation are as follows: (1) to observe the variations in soybean seedling growth under BPA stress and identify the differentially expressed genes and their associated pathways in response to BPA; (2) to reveal the response of endophytic bacterial communities in the leaves and roots of soybean seedlings to BPA; and (3) to elucidate the mechanism underlying the physiological changes in soybean.

## Results

### Effects of BPA on physiological indices of soybean

**Figure 1:**
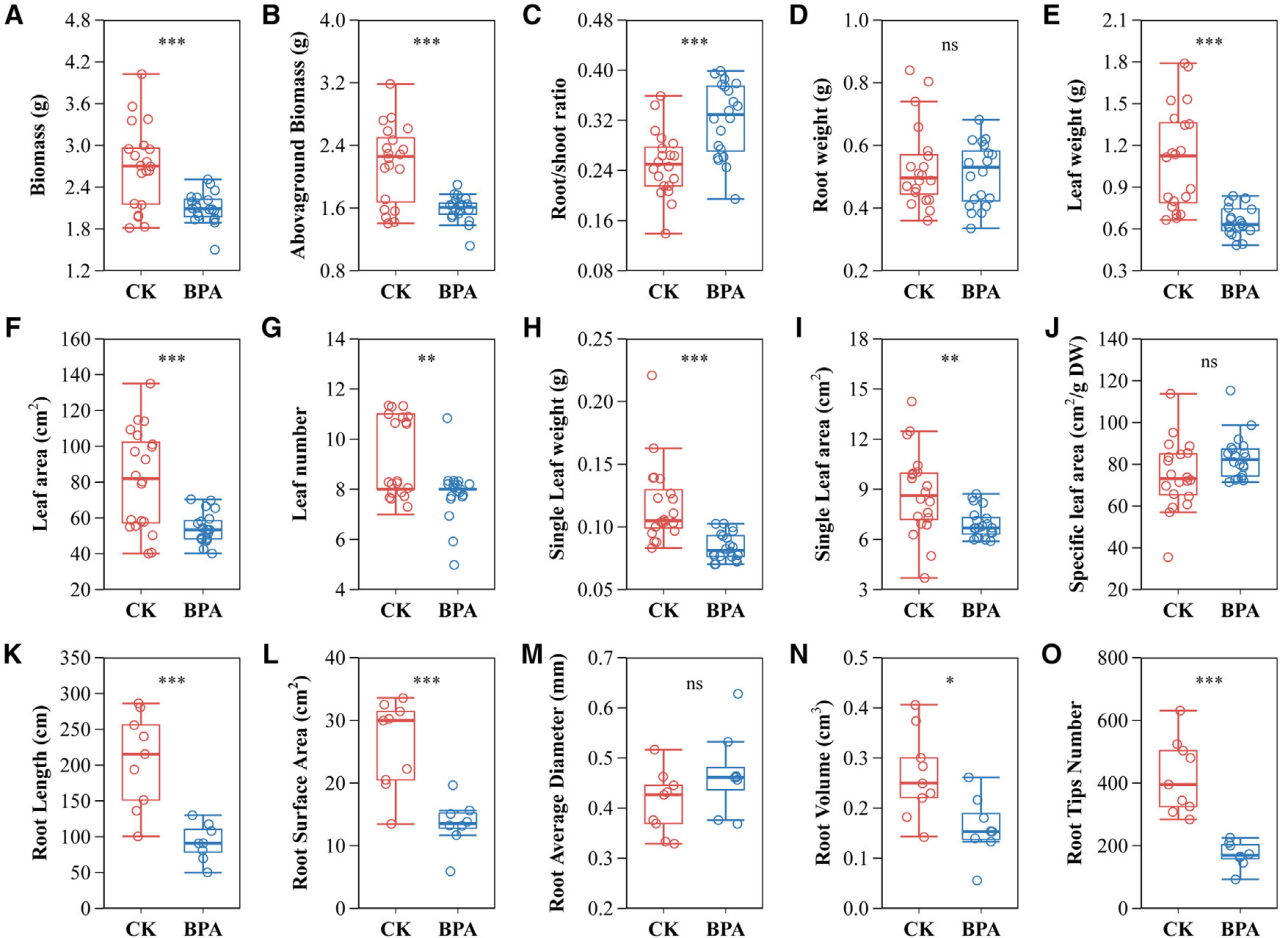
Effect of BPA on on the physiological indicators of soybeans Effect of BPA on soybean biomass (g) (A), aboveground biomass (g) (B), root shoot ratios (C), root weight (g) (D), leaf weight (g) (E), leaf area (cm^2^) (F), leaf number (G), single leaf weight (g) (H), single leaf area (cm^2^) (I), specific leaf area (cm^2^/g DW (dry weight)) (J), root length (cm) (K), root surface area (cm^2^) (L), root average diameter (mm) (M), root volume (cm^3^) (N), and root tip number (O) ∗ Represents *p* < 0.05, ∗∗ indicates *p* < 0.01, ∗∗∗ indicates *p* < 0.001. (A–J: *n* = 20; K–O: *n* = 9 (CK), and 8 (BPA)).

Exposure to BPA significantly diminished the biomass and aboveground biomass of soybean seedlings by 22.2% and 26.6%, respectively, in comparison with the control group (CK) (2.7 ± 0.6 g and 2.2 ± 0.5 g) (Figures 1A and 1B). BPA treatment did not significantly impact root weight, but it did lead to a 28.1% increase in the root/shoot ratio (Figures 1C and 1D). BPA treatment caused a significant decrease in leaf weight, leaf area, leaf number, single leaf weight, and single leaf area of soybean seedlings by 40.6%, 33.4%, 15.6%, 28.6%, and 20.5%, respectively, with no significant alteration in the specific leaf area (Figures 1E–1J). Moreover, the application of BPA resulted in a significant reduction in the root length, root surface area, root volume, and root tip number of soybean seedlings by 55.3%, 48.0%, 39.2%, and 59.2%, respectively, and the average root diameter increased by 14.3% without significance (Figure 1K–1O).

### Effects of BPA on the soybean transcriptome

**Figure 2:**
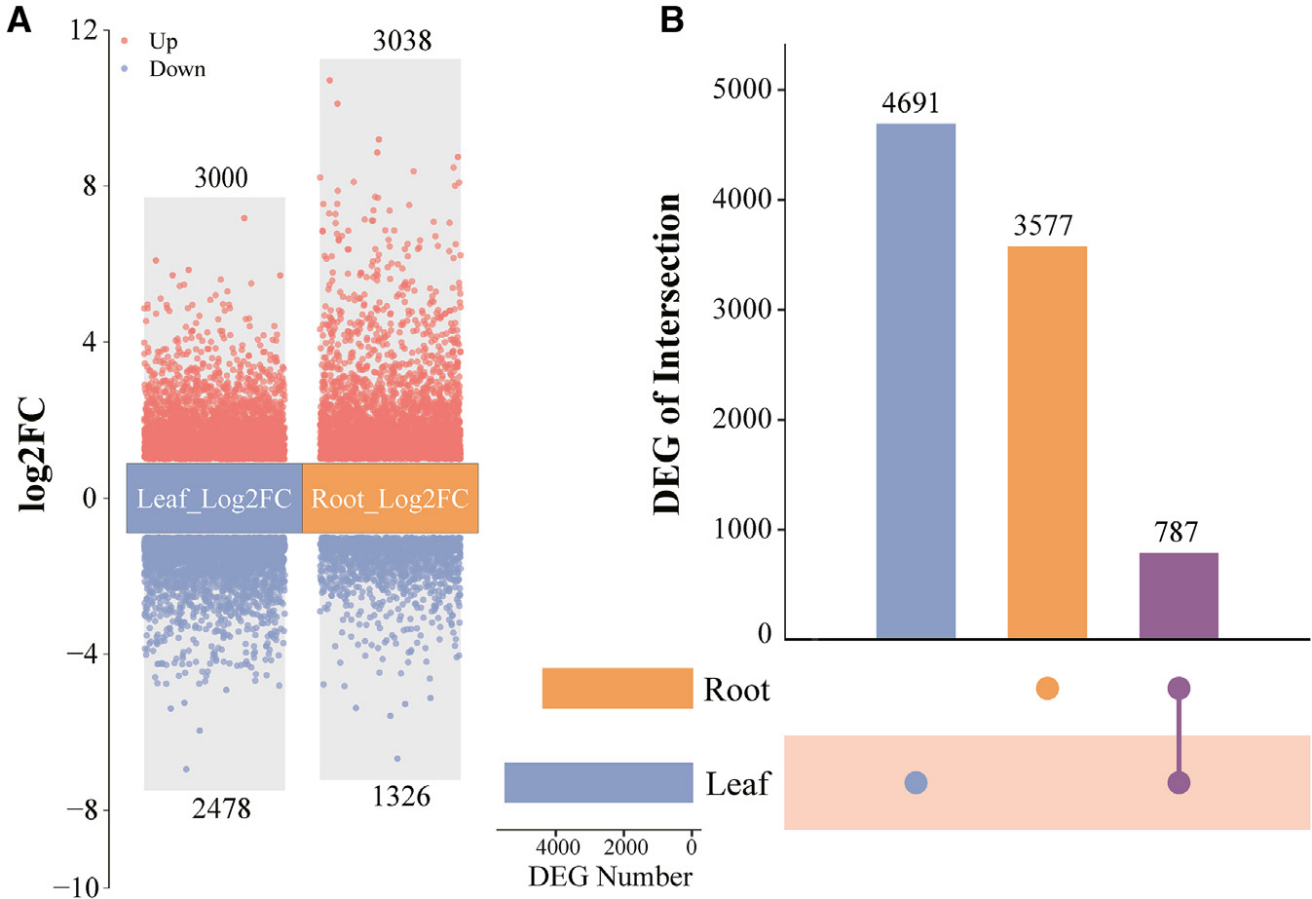
Volcano and UpSet plot analysis of differentially expressed genes in leaves and roots of soybean seedlings under BPA stress The volcano plot illustrates the differential status of differentially expressed genes (DEGs) in leaves and roots of soybean seedlings under BPA stress, using a threshold of a |log2-fold change| of ≥1 and an FDR <0.5 (A) The UpSet plot visualizes the number of differential DEGs among the leaves and roots of soybean seedlings (B) The numbers above the columns represent the number of DEGs.

BPA treatment resulted in a gene expression pattern in soybean seedlings, revealing 5478 DEGs in the leaves (3000 upregulated and 2478 downregulated). In the roots, 4364 DEGs were detected, with 3038 upregulated and 1326 downregulated (Figure 2A, Tables S1 and S2). Among these, 787 genes were consistently expressed in all soybean leaves and roots. At the same time, 4691 and 3577 unique DEGs were identified in the leaves and roots, respectively (Figure 2B). These findings highlight the activation of multiple genes in soybeans in response to BPA stress, with variations observed in the reactions between leaves and roots.

**Figure 3:**
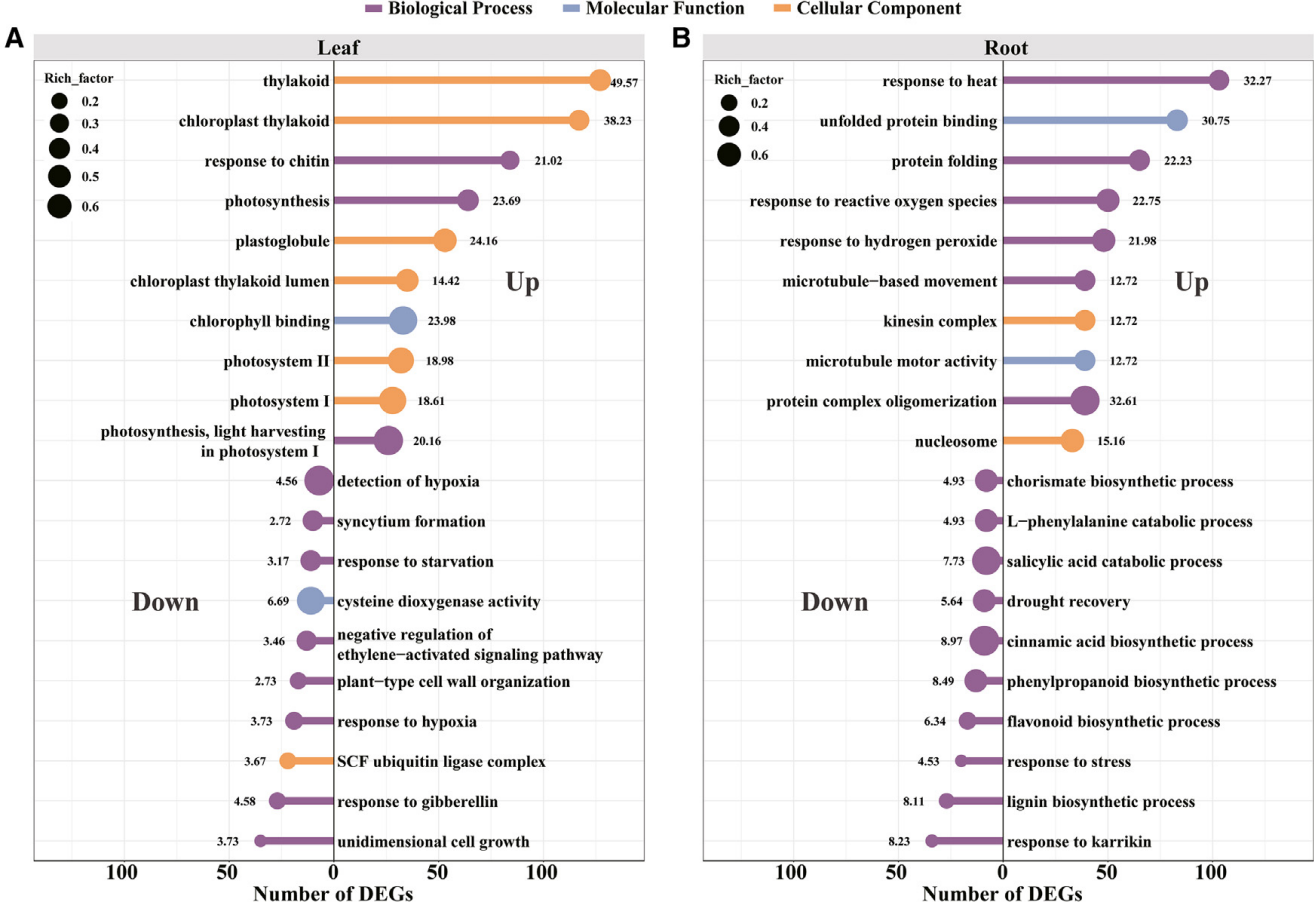
Enrichment analysis of Gene Ontology (GO) identified the top 10 significant terms enriched in leaves and roots of soybean seedlings under BPA stress Enrichment analysis of Gene Ontology (GO) identified the top 10 significant terms (adjusted *p* value <0.05, Benjamini–Hochberg correction) enriched in upregulated and downregulated differentially expressed genes (DEGs) in leaves (A) and roots (B) of soybean seedlings under BPA stress The color of the lollipop indicates the category of GO terms. The value next to each circle represents -log10 (*p* value), where a larger -log10 (*p* value) means a more reliable enrichment significance of the DEGs in the GO terms. The size of each circle represents the rich factor in the GO terms, with larger circles indicating a higher degree of enrichment of the differential genes enriched in the GO terms.

A GO enrichment analysis was executed to unravel the distinct biological functions of DEGs. Figure 3 features the top 10 significantly enriched GO terms associated with the upregulated and downregulated DEGs in both the leaves and roots. The leaf contained 4768 differentially expressed genes (DEGs) annotated across 204 GO terms. The upregulated DEGs were predominantly related to “thylakoid” (GO: 0009579, CC), “chloroplast thylakoid” (GO: 0009534, CC), and “response to chitin” (GO: 0010200, CC). Conversely, “unidimensional cell growth” (GO: 0009826, BP), “response to gibberellin” (GO: 0009739, BP), and “SCF ubiquitin ligase complex” (GO: 0019005, CC) were the prevailing subgroups among the downregulated DEGs (Figure 3A). Notably, 17 downregulated DEGs were annotated to the “plant-type cell wall organization” term (GO: 0009664, BP) (Figure 3A and Table S3). Overall, the downregulated genes predominantly participate in pathways related to growth.

In the root sample, the upregulated DEGs (2603) were primarily associated with enriched GO terms such as “response to heat” (GO: 0009408, BP), “unfolded protein binding” (GO: 0051082, MF), and “protein folding” (GO: 0006457, BP) (Figure 3B). Moreover, these enriched upregulated DEGs encapsulated numerous hormone and stress-related biological processes, including “response to reactive oxygen species”, “response to hydrogen peroxide”, “response to chitin”, “cellular response to heat”, “response to brassinosteroid”, “cellular response to molecule of bacterial origin”, and “regulation of defense response” (Table S3). The downregulated DEGs (1157) were significantly enriched in “response to karrikin” (GO: 0080167, BP), “lignin biosynthetic process” (GO: 0009809, BP), and “response to stress” (GO: 0006950, BP) (Figure 3B). Furthermore, 24 downregulated DEGs were annotated to two additional biological processes, namely, “lateral root formation” (GO: 0010311) and “xylem development” (GO: 0010089) (Table S3). Overall, the upregulated genes in roots primarily participated in stress response and protein synthesis pathways, while the downregulated genes largely contributed to growth-related pathways.

### Effects of BPA on the endophytic community in soybean

**Figure 4:**
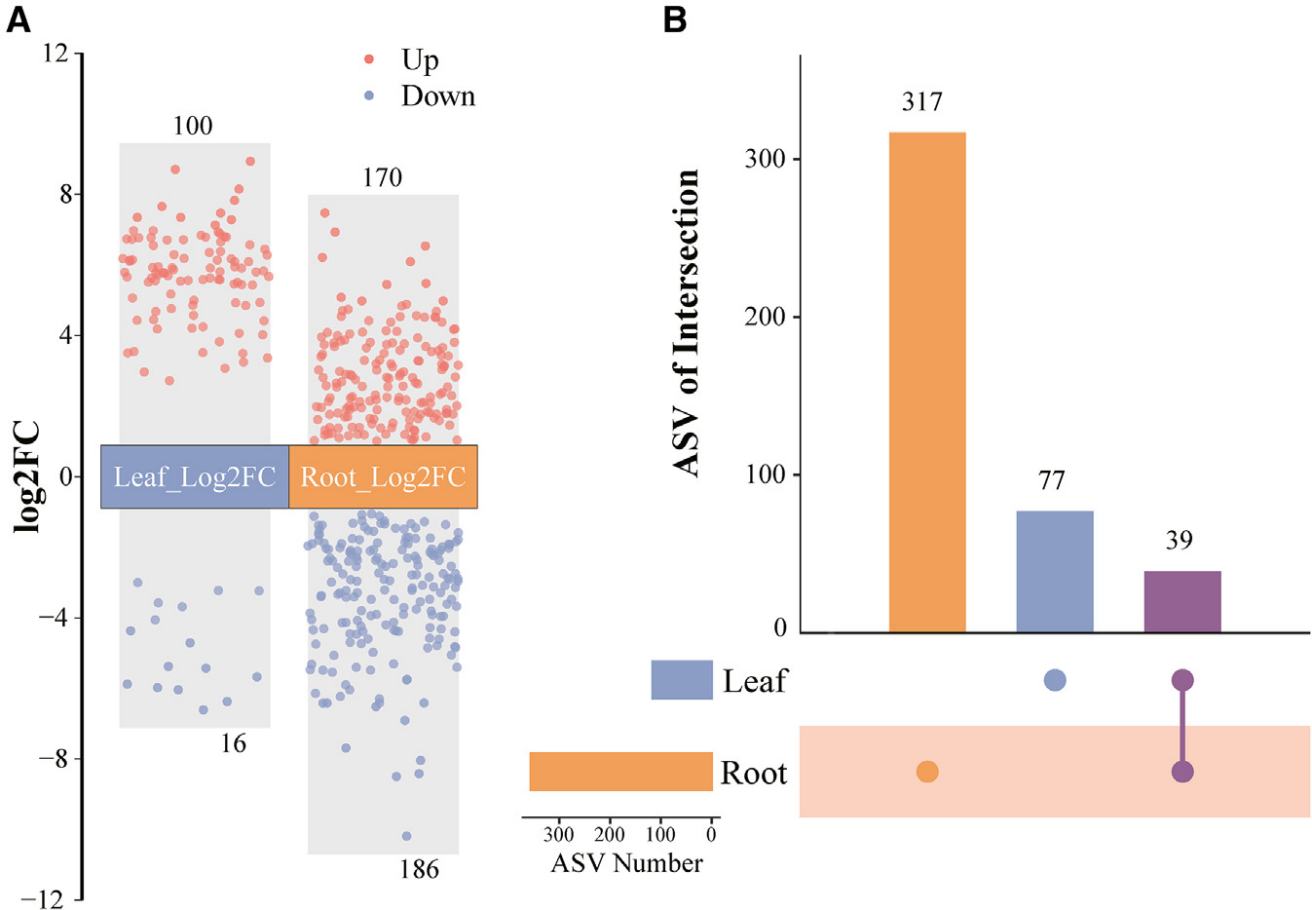
Differential expression analysis of Amplicon Sequence Variants (ASVs) in soybean seedlings under BPA stress illustrated by volcano and UpSet plots The volcano plot illustrates the differential status of Amplicon Sequence Variants (ASVs) in leaves and roots of soybean seedlings under BPA stress, using a threshold of a |log2-fold change| of ≥1 and an FDR <0.5 (A). The UpSet plot visualizes the number of differential ASVs among the leaves and roots of soybean seedlings (B). The numbers above the columns represent the number of differential ASVs

After amplifying and sequencing the 16S rRNA gene, 3454 ASVs were identified (Table S4). Differential analysis results showed that 116 ASVs were identified in the leaves of soybean seedlings, with 100 exhibiting upregulation and 16 demonstrating downregulation. Additionally, 356 (170 upregulated and 186 downregulated) differential ASVs were ascertained in the roots of soybean seedlings (Figure 4A and Table S5). Moreover, 39 differential ASVs were shared between the leaves and roots, while a distinct set of 317 differential ASVs was exclusively detected in the roots (Figure 4B).

**Figure 5:**
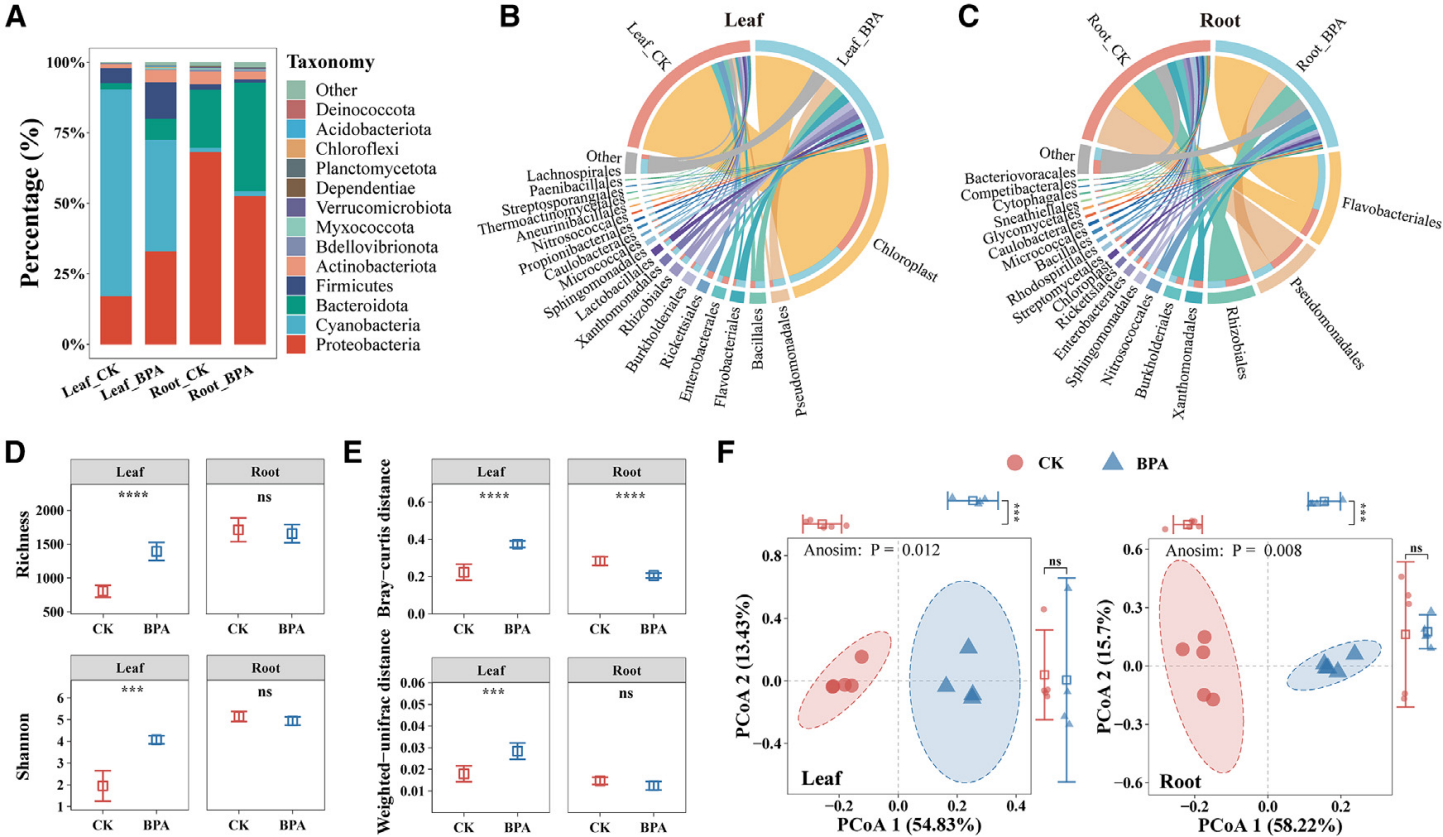
The top 10 bacterial phyla had the highest average relative abundance, and the top 20 orders had the highest average relative abundance across the leaf and root of soybean seedlings The top 10 bacterial phyla had the highest average relative abundance (A), and the top 20 orders had the highest average relative abundance across the leaf (B) and root (C) of soybean seedlings. The endophytic bacterial richness and Simpson diversity of leaves and roots of soybean seedlings (mean ± ci) (D). Bray‒Curtis distance and weighted UniFrac distance of leaves and roots of soybean seedlings (mean ± ci) (E). Principal coordinate analysis (PCoA) representing the endophytic community from leaves and roots of soybean seedlings with BPA treatment based on Bray‒Curtis distance (Anosim test and permutation test by 999) (F). Leaf_CK and Root_CK represent the leaves and roots of soybean seedlings in the control treatment, respectively. Leaf_BPA and Root_BPA represent the leaves and roots of soybean seedlings in the BPA (50 mg/L) treatment, respectively. ∗*p* < 0.05, ∗∗*p* < 0.01, ∗∗∗*p* < 0.001. *n* = 5; n (Leaf_BPA) = 4

The introduction of BPA resulted in changes to the composition of endophytic bacteria within the soybean community, with variations observed across different plant parts. Treatment with BPA decreased the relative abundance of Cyanobacteria from 73.40% to 39.58% within the leaves. Within Cyanobacteria, the relative abundance of Chloroplast was observed to have declined by 39.55% compared to the control group (73.40%). The abundance of Proteobacteria (from 16.99% to 32.93%) increased, and Pseudomonadales exhibited a relative abundance increase of 6.25%. Firmicutes increased abundance, rising from 5.43% to 12.92%, with Bacillales showing a relative abundance increase of 3.50% (Figures 5A and 5B). The dominant phylum in the roots, Proteobacteria, experienced a decrease in relative abundance from 68.14% to 52.62%. BPA stress caused a reduction in the relative abundance of Pseudomonadales (from 25.56% to 14.16%), Rhizobiales (from 16.26% to 11.94%), and Xanthomonadales (from 6.06% to 3.90%) in Proteobacteria. In contrast, there was an increase in the relative abundance of Bacteroidota from 20.66% to 38.44%, primarily attributed to a significant surge in the relative abundance of Flavobacteriales from 19.04% to 37.36% (Figures 5A and 5C).

The presence of BPA resulted in changes in the diversity of endophytic bacteria in soybean seedlings. The richness and Shannon indices of endophytic bacteria in the leaves experienced significant increases of 72.76% and 108.42%, respectively, while no significant changes were observed in the roots compared to the control group (Figure 5D). Exposure to BPA had a significant impact on the beta diversity of internal microbial communities in different parts of the soybean seedlings. Specifically, our analyses revealed significant increases of 67.39% and 58.60% in Bray‒Curtis and weighted UniFrac distances in the leaves, respectively. Conversely, the roots exhibited a substantial reduction of 27.64% in Bray‒Curtis distance, whereas the weighted UniFrac distance remained essentially unchanged (Figure 5E). Additionally, PCoA and ANOSIM indicated significant alterations in the composition of internal microbial communities in response to BPA exposure, with *p* values of 0.012 and 0.008 recorded for the respective plant parts (Figure 5F).

### Correlation between endophytes and the transcriptome of soybean

**Figure 6:**
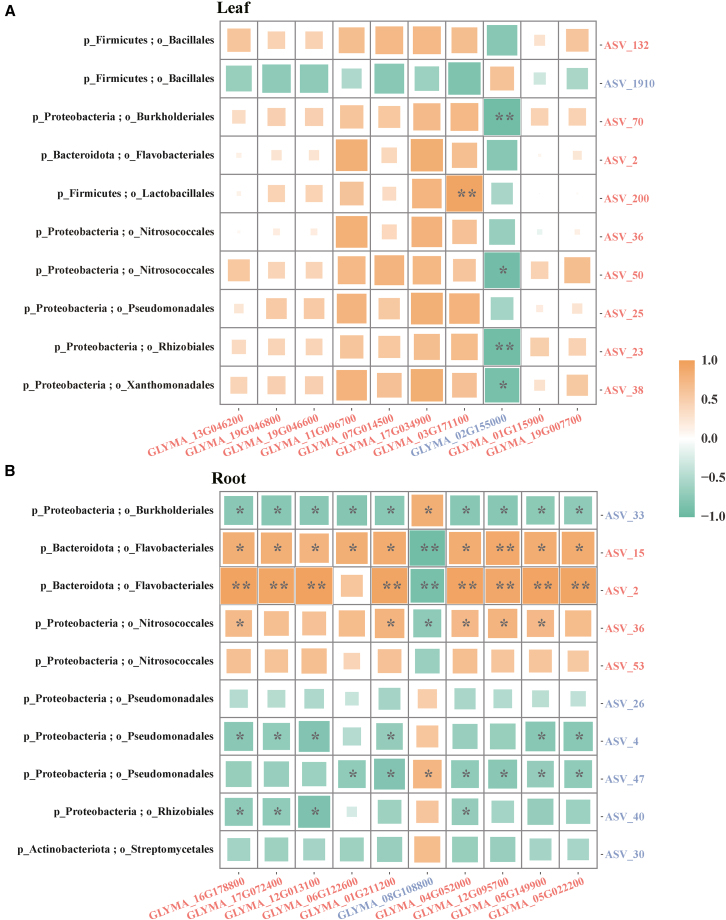
Correlation analysis of Top 10 amplicon sequence variants (ASVs) and differentially expressed genes (DEGs) in soybean seedlings under BPA stress Correlation analysis (Spearman) of BPA treatment and control group by the top 10 (based on the relative abundance and relative expression sort) amplicon sequence variants (ASVs) and differentially expressed genes (DEGs) of leaves (A) and roots (B) of soybean seedlings, and the threshold of a |log2-fold change| of ≥1 and a *p* value <0.5. Each row corresponds to an ASV, and the column corresponds to a DEG. The color and asterisks of each cell at the row-column intersection indicate the corresponding correlation coefficient and adjusted *p* value (Benjamini–Hochberg correction, ∗*p* < 0.05, ∗∗*p* < 0.01) between the ASV and the DEG. The color from orange to green represents r values from 1 to −1. The red word indicates that the expression of the DEGs or the relative abundance of ASVs is upregulated compared with the control group, while the blue word means downregulation. The black words mark the species annotation information of ASVs (p: phylum, o: order)

A DESeq2 differential analysis was conducted to assess the impact of BPA treatment by comparing the BPA treatment group with the control group. The top 10 differential ASVs and DEGs in the leaf and root of soybean seedlings were selected for Spearman correlation analysis, focusing on relative abundance and expression level. The findings indicated a significant positive correlation between the expression level of *heat shock cognate 70 kDa protein 2* (*LOC100798307, GLYMA_03G171100*) and the relative abundance of ASV_200 (Lactobacillales, r = 0.93) in the leaves. Conversely, the downregulated gene *GLYMA_02G155000*, which encodes the *AAA+ ATPase domain-containing protein*, exhibited significant negative correlations with ASV_70 (Burkholderiales, r = −0.93), ASV_50 (Nitrosococcales, r = −0.92), ASV_23 (Rhizobiales, r = −0.93), and ASV_38 (Xanthomonadales, r = −0.88) in the samples (Figure 6A and Table S6). A significant correlation was observed between ASVs and DEGs in the root. Specifically, Flavobacteriales (ASV_2, ASV_15), Nitrosococcales (ASV_36), Burkholderiales (ASV_33), and Pseudomonadales (ASV_4, ASV_47) exhibited significant correlations with at least six of the top ten DEGs (Figure 6B). A significant negative correlation was observed for the upregulation of genes (*HSP70*, *GLYMA_17G072400*) encoding *heat shock 70 kDa protein-like* proteins and the reduced relative abundance of *Proteobacteria* (ASV_33, ASV_4, ASV_40), while a significant positive correlation was observed with the increased relative abundance of Bacteroidota (ASV_2, ASV_15) (Figure 6B).

Furthermore, a significant correlation existed between the relative abundance of ASVs and the expression of DEGs in the top 5 (sorted according to fold change values of upregulated and downregulated) within the leaf and root (Figure S3). Among these, the downregulated *ARG7 auxin responsive family protein* (*LOC100500265*, *GLYMA_12G035700*) in the leaves showed significant positive correlations with ASV_1301 (Acetobacterales, r = 0.86), ASV_2677 (Enterobacterales, r = 0.79), and ASV_1082 (Sphingomonadales, r = 0.79) but exhibited significant negative correlations with ASV_67 (Sneathiellales, r = −0.86), ASV_324 (Xanthomonadales, r = −0.86), ASV_231 (r = −0.86), and Bacteroidota (ASV_422, ASV_431) under BPA stress (Figure S3A and Table S6). Within the roots, the upregulation of DEGs within the top 5 significantly correlated with the top 10 ASVs of high variability under BPA stress. Furthermore, the gene (*LOC100796311*, *GLYMA_09G284700*) encoding *peroxidase 5*, which was downregulated, displayed a significant negative correlation with Nitrosococcales (ASV_93, ASV_289, ASV_462) (Figure S3B).

## Discussion

### Effects of BPA on physiological indices and the transcriptome of soybean seedlings

The findings of this study indicate that the continuous introduction of BPA into the soil environment has a significant detrimental effect on soybean growth, which aligns with previous research.^8^^,^^16^^,^^17^^,^^43^ The adverse impact of BPA on leaf-related indices and biomass (Figure 1), aligning with previous studies that have demonstrated the inhibitory effects of high BPA doses on leaf in soybeans.^12^^,^^44^ The study showed that soybean roots were significantly inhibited (Figure 1O), which is consistent with previous studies that found BPA caused root tip cell death in soybeans.^17^ In the present study, it was observed that the application of BPA resulted in a significant decrease in the number of leaves, the weight of a single leaf, and the area of a single leaf, thereby implying a constraint on the overall process of photosynthesis (Figures 1G–1I). Simultaneously, a significant reduction in the number of root tips was observed (Figure 1O), indicating an impediment in root elongation and a diminished ability to acquire nutrients.^10^ The observed outcome can be ascribed to the excessive generation of reactive oxygen species (ROS) caused by BPA, resulting in damage to the ultrastructure of mitochondria, inhibition of the activity of crucial energy-production enzymes, and a decrease in ATP synthesis and cell death in roots.^17^^,^^45^^,^^46^ Consequently, we posit that the diminished photosynthetic capacity and impaired nutrient uptake capability are likely the principal determinants leading to the reduction in total biomass.

This phenomenon can be ascribed to modifications in the gene expression profile of soybeans. Our investigation revealed that exposure to BPA stress, similar to other stresses, such as drought^47^^,^^48^ and herbicide exposure,^49^ induces changes in the gene expression patterns of soybean seedling leaves and roots during regular developmental processes (Figure 2). This finding is consistent with the research findings of Tian et al.^21^ and Xiang et al.,^22^ which demonstrated that BPA has an impact on gene expression. Previous studies have indicated that exposure to stressors (salt stress, water stress) can lead to gene expression changes in the biological processes of plant cell wall tissue.^50^^,^^51^^,^^52^ In our study, a GO enrichment analysis revealed that the significantly downregulated genes within the leaves of soybean seedlings were significantly enriched in terms related to the “response to gibberellin”, “unidimensional cell growth”, and “plant-type cell wall organization” (Figure 3A). These findings additionally corroborated the significant reduction in the area and the number of leaves under BPA stress (Figures 1E–1I).

Furthermore, the downregulated DEGs in the roots of soybean seedlings exhibited significant enrichment in terms related to “xylem development”, “lateral root formation”, and “regulation of meristem growth”. These DEGs were also implicated in multiple pathways associated with metabolite biosynthesis (Figures 3B and S4, Tables S3 and S7). This finding is consistent with the adverse impact on the expression of genes and metabolites associated with cell wall synthesis in *Solanum nigrum* under cadmium stress.^53^ Previous research has demonstrated that the exposure of plants to stressors leads to the suppression of gene expression related to cell walls, resulting in the inhibition of root growth.^54^^,^^55^ Our study found that soybean root growth inhibition under BPA-induced pressure significantly inhibited root tips, consistent with the transcriptome results (Figures 1K–1O). Consequently, the reduction in biomass is associated with the downregulation of leaf and root growth genes (Figure 1).

### Effects of BPA on the composition and diversity of the endophytic community in soybean

Our study revealed that continuous exposure to BPA significantly impacted the structure and composition of the endophyte community in soybean seedlings (Figures 4, 5 and S5). This finding aligns with previous studies demonstrating that BPA stress alters microbial communities.^26^^,^^30^^,^^56^ Specifically, we observed an increase in the dominant phylum Proteobacteria in leaves but a decrease in the roots. Additionally, Bacteroidetes experienced a significant increase after BPA treatment, and the distribution of predominant bacteria in soybeans varied among different parts (Figure 5A). Huang et al.^29^ discovered that *Pseudomonas* and *Sphingomonas* constituted more than 73% of the original bacterial community after BPA treatment. Our research demonstrated that the relative abundance of Sphingomonadales exhibited an increase in both leaves and roots. However, the abundance of Pseudomonadales increased in leaves but decreased in roots (Figures 5B and 5C). Sphingomonadales can break down pollutants like BPA,^57^ increasing their presence in leaves and roots. This rise in leaves may be due to reduced BPA levels allowing Pseudomonadales to adapt,^58^ while the decrease in roots might be caused by high BPA concentrations limiting their growth and competitiveness.

Simultaneously, our investigation ascertained that the presence of BPA augmented the alpha diversity of endophytes in leaves (Figure 5D). In contrast, Huang et al.^59^ conducted research revealing that the presence of di-(2-ethylhexyl) phthalate contamination considerably diminished bacterial community diversities in both the rhizosphere and rhizoplane of maize. This inconsistency could be attributed to the migration of BPA into soybean seedling leaves via the root system,^60^ consequently attracting specific bacteria capable of utilizing BPA as a growth substrate.^57^^,^^58^^,^^61^ However, our experiment did not identify significant changes in the abundance and diversity of endophytes in roots (Figure 5D). This lack of significant change may be attributed to root system endophytes receiving inputs and supplements from soil microorganisms,^62^^,^^63^ thus maintaining a certain level of stability. Notably, roots exhibited the highest number of differential ASVs (Figure 4B), which can be attributed to their direct exposure to BPA in the soil. This direct exposure enhances the responsiveness of endophytic bacteria residing within the roots to BPA.

### The mechanism of BPA affecting the growth of soybean

The results of our study demonstrate a noteworthy association between the microorganisms and transcriptome of soybean seedlings subjected to BPA stress. In particular, a strong correlation was observed between the ASVs and the DEGs that exhibited the most significant alterations under BPA stress (Figures 6 and S3). Extensive scientific investigations have indicated that plants can enlist advantageous microbiota to regulate their growth and developmental processes.^62^^,^^63^ Previous studies have shown that inoculating plant growth-promoting bacteria can modulate the expression of host genes.^35^ Simultaneously, endophytes have been discovered to alleviate the adverse effects of pollutant stress on plants,^61^^,^^64^ potentially altering plant gene expression.

Our investigation revealed a strong correlation between Proteobacteria and Bacteroidota and the gene expression levels in soybeans (Figures 6 and S3). Previous research has highlighted the positive contributions of genera such as *Pseudomonas* sp. and *Bacillus* sp. from the Proteobacteria phylum, as well as several members of the Bacteroidota phylum (such as *Flavobacterium* sp., *Sphingobacterium* sp.), to plant growth.^65^^,^^66^^,^^67^^,^^68^ Furthermore, our research reveals that the relative abundance of the orders Pseudomonadales, Flavobacteriales, and Bacillales underwent changes under BPA treatment in soybean endophytes (Figure b,c). Studies have demonstrated a positive correlation between beneficial plant microorganisms and specific plant metabolites during periods of stress.^69^ Additionally, our study observed significant alterations in the gene encoding cytochrome P450 (CYP) in soybean seedlings under BPA stress, with an observed upregulation in both the leaves and roots (Table S2). Previous studies suggest that CYP enzymes, functioning as monooxygenases, can degrade BPA^70^ and that bacteria can achieve this degradation by producing CYP.^58^^,^^71^

The enrichment analysis conducted in this study revealed a notable enrichment of upregulated genes in both leaves and roots, specifically in stress response pathways such as “response to stress” and “response to heat” (Figure 3 and Table S3), which aligns with previous investigations.^49^^,^^72^ Additionally, the upregulated genes identified in the roots were found to be linked to protein processing (Figures 3 and S4). Prior research has documented that soybeans experience growth inhibition due to oxidative stress induced by BPA.^45^ Consistent with this observation, our investigation demonstrated that the upregulated genes in soybeans were significantly enriched in the biological process of “response to hydrogen peroxide”, and we identified an upregulation in the expression level of 21 genes encoding glutathione S-transferase (GST) (Figure 3 and Table S2). GST has been documented to eliminate peroxide by conjugating it with glutathione and plays crucial roles in catalytic and nonenzymatic functions during the plant stress response.^73^^,^^74^^,^^75^ These findings suggest that soybeans can withstand BPA stress by activating defense and stress response mechanisms and enhancing their detoxification capabilities.

It was observed that the upregulated gene (*GLYMA_10G234700*) encoding cytokinin hydroxylase in leaves and the upregulated gene (*GLYMA_17G072400*) named *HSP70* in roots exhibited significant associations with both Proteobacteria and Bacteroidota (Figures 6 and S3). Previous studies have demonstrated that inoculating plant-growth-promoting bacteria affects the antioxidant defense system and the expression of genes related to abiotic stress, resulting in stress alleviation.^34^^,^^37^ Moreover, it has been ascertained that Hsp70 exerts a significant influence on plant stress and adversity responses, whereas cytokinin hydroxylase regulates the degradation, level, and signal transduction of cytokinins, thereby impacting plant growth and development.^76^^,^^77^ Furthermore, investigations have demonstrated that endophytic *Bacillus* spp. elicit the upregulation of defense genes in maize,^33^ and a study conducted by Begum et al.^78^ has corroborated that plant growth-promoting bacteria can improve the expression of the HSP70 gene under stressful conditions. The results indicate that soybean endophytes may respond to BPA stress by upregulating stress resistance gene expression.

Consequently, our study has demonstrated that the downregulated genes in the leaves and roots of soybean seedlings are closely connected to growth regulation. Simultaneously, endophytes have the potential to influence gene expression in soybean seedlings. This investigation provides valuable insights into the alterations occurring in the transcriptome and endophyte community of soybean seedlings when subjected to BPA-induced stress. This finding deepens our understanding of the impact of BPA on crop growth, especially soybeans, from the perspective of endophytes and transcriptomes, thereby providing a scientific basis for assessing the implications of BPA on agricultural yield.

### Limitations of the study

This study verified that the continuous application of BPA in soil has a significantly negative impact on the growth of soybean seedlings. Furthermore, BPA stress leads to alterations in gene expression within soybean seedlings, characterized by the upregulation of stress-related genes and the downregulation of genes associated with growth-related pathways, ultimately impeding seedling development. Moreover, the continuous application of BPA altered the composition of endophytic bacteria in soybeans, remarkably increasing the abundance and diversity of endophytic bacteria in seedling leaves. The correlation analysis between soybean endophytes and the transcriptome elucidated that the introduction of BPA might influence the gene expression of soybean seedlings by altering endophytes. Above all, the findings of this study indicate that the stress induced by BPA hindered the growth of plants through the modulation of gene expression associated with growth. This establishes a scientific basis for assessing the effects of BPA on crop growth and integrating BPA level assessments and microbial health into soil management strategies.

Unfortunately, this investigation does not explicitly establish a direct correlation between the transcriptome, microbiome, and growth parameters. Future research can focus on understanding the molecular pathways through which BPA affects soybean growth and microbial communities.

## Resource availability

### Lead contact

Further information and requests for resources and reagents should be directed to and will be fulfilled by the lead contact, Chuansheng Wu (wwccss521@163.com).

### Materials availability

This study did not generate new materials.

### Data and code availability

• All data reported in this paper will be shared by the lead contact upon request.
• This paper does not report original code.
• Any additional information required to reanalyze the data reported in this paper is available from the lead contact upon request.

## Acknowledgments

This work was supported by the Natural Science Foundation of Universities of Anhui Province for Distinguished Young Project (2022AH020081), the Opening Project of Anhui Province Key Laboratory of Environmental Hormone and Reproduction, 10.13039/501100012404Fuyang Normal University (No. FSKFKT012), the Innovative Research Team in University of Anhui Province (2022AH010079), the Natural Science Research Program of Anhui Higher Education Institutions (2022AH05134, 2022AH051328, 2024AH051458), the 10.13039/501100001809National Natural Science Foundation of China (41975147), the Young and Middle-aged Academic and Technical Leaders Reserve Talents Program in Yunnan Province (202305AC160090), and the Biological and Medical Sciences of Applied Summit Nurturing Disciplines in Anhui Province (Anhui Education Secretary Department [2023]13).

## Author contributions

Conceptualization, C.W. and J.T.; data curation, C.H.; formal analysis, C.W. and K.W.; funding acquisition, C.W., J.T., X.L., Y.M., and Y.L.; investigation, C.W.; methodology, C.W.; resources, C.W. and J.T.; visualization, C.W., K.W., and C.H.; writing – original draft, K.W., N.Z., M.Y., W.T., and Y.Z.; writing – review and editing, K.W., N.Z., C.W., L.Z., X.L., J.T., Y.M., Y.L., and Y.L.

## Declaration of interests

The authors declare no competing interests.

## STAR★Methods

### Key resources table

**Table:** 

REAGENT or RESOURCE	SOURCE	IDENTIFIER
**Chemicals, peptides, and recombinant proteins**		

Bisphenol A (BPA)	Aladdin	CAS: 80-05-7

**Critical commercial assays**		

TruSeqTM RNA Sample Preparation Kit	Illumina platform	N/A
HiPureSoilDNA extraction kit	Magen	N/A

**Software and algorithms**		

RStudio	N/A	https://posit.co/download/rstudio-desktop/
R	N/A	https://cran.r-project.org/
USEARCH v11	Edgar	https://www.drive5.com/usearch/manual/cmd_otutab_rare.html

**Other**		

Electronic balance	Sartorius	https://www.sartorius17.cn/
Root analysis system	Regent Instruments	https://regentinstruments.com/
NanoDrop2000 spectrophotometer	Thermo Scientific	https://www.thermofisher.cn/cn/zh/home/industrial/spectroscopy-elemental-isotope-analysis/molecular-spectroscopy/uv-vis-spectrophotometry/instruments/nanodrop.html

### Experimental model and study participant details

This study does not involve animal experiments.

### Method details

#### Pot experimental design

The greenhouse pot experiment was conducted at Fuyang Normal University (32°53′N, 115°46′E; Fuyang, China) laboratory facilities, spanning from April 23, 2021, to May 29, 2021. During the cultivation period, the greenhouse conditions were maintained, with a daily lighting time of 14 h, an average temperature of 28.0°C, and a mean humidity of 61.6% (Figure S1). The soybeans (*Glycine max*) used in the experiment were selected based on intact seed coats and uniform size and color. The investigation was conducted using a set of eight 8 L pots, eight arranged on a single layer of a culture rack measuring (dimensions: 120 cm length, 50 cm width, 70 cm height).

To previous research on the effects of BPA on plants, a concentration of 50 mg/L BPA was selected for this experiment.^17^^,^^79^ Sixty pots were prepared for the two treatment groups (CK (Control Group), BPA-free; BPA, with an added concentration of 50 mg/L BPA). Each group consisted of thirty pots, and within each pot, twelve soybeans were planted at a uniform depth of 3 cm. BPA, acquired from Shanghai Aladdin Biochemical Technology (>99.0% (GC)), was grind and applied at a mass of 400 mg, taking into account the actual volume capacity of the pots (8 L). BPA was weighed using an electronic balance (BSA124S, Sartorius, Shanghai, China) and dissolved in 1 L of deionized water, with each treatment group irrigated with either deionized water or the BPA solution. According to prior research, it was determined that the half-life of the solution when applied to soil was seven days.^80^^,^^81^ Consequently, to maintain a prolonged impact, a BPA solution with a half-concentration was administered weekly. Notably, owing to the retention of soil particles, BPA is retained on the soil surface, forming white particles. Therefore, the addition of BPA can only be used as a specific treatment in this study.

#### Measurements of plant growth parameters and sample collection

The duration of the pot culture experiment’s monitoring phase spanned approximately five weeks. Once the actual leaves of the soybean plants had emerged, seedlings exhibiting disparate growth rates were eliminated to ensure consistent growth within each treatment, with a total of 20 plants being retained per treatment. On May 17, 2021, samples were gathered because the intricate nature of the root system during the later seedling stage hindered the determination of root system traits. The soybean seedlings were separated and subjected to drying to ascertain the biomass of different tissues, including roots, stems, and leaves. Leaf and root parameters, including leaf area and root characteristics (length, surface area, average diameter, volume, and tip count), were assessed using a root analysis system (WinRHIZO Pro 2019, Regent Instruments, Quebec, Canada). This was done after scanning the plane images of leaves and roots with an EPSON scanner, yielding 9 and 8 complete soybean root systems from 20 soybean seedlings in the CK and BPA groups, respectively. The collection of soybean seedling leaves and roots was performed on May 29, 2021, following the sampling methodology described by Wu et al.^82^ All specimens were promptly preserved in liquid nitrogen and kept at −80°C for subsequent examinations of endophytes and transcriptomes, with each trial employing five biological duplicates.

#### Transcriptome data acquisition and processing

Soybean tissue samples were subjected to total RNA extraction and agarose gel electrophoresis to assess RNA sample integrity. RNA purity and concentration were determined using a NanoDrop Spectrophotometer (Nanodrop 2000), while the RNA integrity number (RIN) value was established using an Agilent 2100 Bioanalyzer. The sequencing library was constructed using the Illumina TruSeqTM RNA Sample Preparation Kit, and sequencing was performed on the Illumina platform (NovaSep 6000 system), resulting in paired-end reads with a read length of 2 × 150 bp.

The quality of raw reads in fastq format was evaluated using FastQC (https://www.bioinformatics.babraham.ac.uk/projects/fastqc/), resulting in clean data in which Q30 bases exceeded 92.63%. All subsequent analyses were conducted on these high-quality clean data. The reference genome (FASTA format) and gene annotation (GTF format) files of *Glycine max*, obtained from the Ensembl database (http://plants.ensembl.org/Glycine_max/Info/Index), were utilized to build a genome index using STAR (https://github.com/alexdobin/STAR). The cleaned reads from each sample were aligned against this reference genome index using STAR, and the resulting read counts mapped to individual genes were obtained. These counts were then used to generate a gene expression matrix through HTSeq (https://github.com/htseq/htseq).

All *Glycine max* transcripts were annotated using various databases, including the Ensembl database, NCBI database (https://www.ncbi.nlm.nih.gov/datasets/genome/GCF_000004515.6/), SoyBase database (https://soybase.org),^83^ Gene Ontology (GO) database (https://geneontology.org/), Joint Genomics Institute (JGI) database (https://phytozome-next.jgi.doe.gov/info/Gmax_Wm82_a4_v1), and Kyoto Encyclopedia of Genes and Genomes (KEGG) database (https://www.genome.jp/kegg/). The annotation files were then consolidated using R for further analysis of differentially expressed genes. Differential expression analyses for the leaves and roots of soybean seedlings were conducted using the DESeq2 R package. The Benjamini‒Hochberg method was employed to calculate the false discovery rate (FDR) to adjust the *p* value. Genes exhibiting |log2-fold change (FC)| ≥ 1 and an FDR <0.05 were designated differentially expressed genes (DEGs) and subsequently subjected to GO and KEGG enrichment analysis using the clusterProfiler (version 4.0.5) package in R (version 4.2.0). The criterion for screening enrichment analysis was a corrected *p* value <0.05 (Benjamini‒Hochberg correction).

#### 16S rRNA gene high-throughput sequencing and data processing

Genomic DNA was extracted from the leaves and roots of soybean seedling samples using the HiPureSoilDNA extraction kit (Magen, Guangzhou, China) according to the manufacturer’s instructions. The V5-V7 region of bacterial 16S rRNA genes was amplified employing bacterial primers 799F (5′- AACMGGATTAGATACCCKG -3') and 1193R (5′- ACGTCATCCCCACCTTCC -3').^84^ The resulting PCR products were separated on a gel, recovered, and quantified using Qubit 3.0. The amplified products were then combined in equal amounts, ligated with sequencing adapters, and utilized for constructing the sequencing library. The sequencing process was performed on the Illumina MiSeq platform using a PE250 strategy.

Illumina MiSeq platform sequencing data were analyzed using USEARCH v11.^85^ Raw sequence data were processed to merge paired-end reads and remove barcode and primer-binding sequences. Quality filtering was performed to obtain an itag with a maximum expected error ratio of 1%. After removing duplicate sequences, unoise3 denoising was applied to achieve single-base precision amplicon sequence variants (ASVs), resulting in 3453 ASVs. A feature table (ASV table) was then normalized using the otutab rare command to equalize all samples to the same number of reads based on the minimum sample size, generating a clean ASV table for downstream analyses. The SILVA database SSU Ref NR 99 release 138.1^86^ was utilized for the taxonomic classification of bacterial sequences, with a confidence threshold of 0.6. The alpha and beta diversity analysis from the ASV table and the construction of the phylogenetic tree were completed using USEARCH v11.

The results of the hierarchical clustering analysis, which utilized the Bray‒Curtis distance, were visually represented using the R package ggtree.^87^ The findings indicated that the sample bearing the L50-3 sample number was anomalous, resulting in the exclusion of data from sample L50_3 in subsequent microbial data analysis (Figure S2). Differential abundance analysis of soybean bacteria was conducted via the R package DESeq2 (version 1.32.0) and visualized with the R packages ggplot2 and UpSetR. The microbial community composition (due to abundant unannotated species information at the family and genus levels, the community composition is shown at the order level) and diversity were depicted using R language-based packages ggplot2 and circlize. We also utilized principal coordinate analysis (PCoA) and an ANOSIM test (based on 999 permutations) to examine differences in bacterial community composition among the different samples using the Bray‒Curtis distance.

### Quantification and statistical analysis

The statistical analyses were performed using R (version 4.2.0) and RStudio. To assess differences in soybean growth indicators between the BPA-treated and control groups, the Student’s t-test was utilized. The psych package (version 2.2.5) in R, employing the Spearman method, was used to calculate correlations between endophytes and the soybean transcriptome. This involved (1) screening the top 10 expression matrices of ASVs and DEGs (with the threshold of |log2-fold change| ≥ 1 and FDR <0.5) from the leaves and roots of soybean seedlings based on relative abundance and expression level for correlation analysis and (2) the ASVs and DEGs in the leaves and roots of soybean seedlings were sorted based on the log2-fold change value, and the top 5 upregulated and downregulated expression matrices were extracted for correlation analysis. The *p*-values were corrected using the Benjamini-Hochberg method. The ggplot2 package was utilized to visualize the graphics.

## Supplemental information

Document S1. Figures S1–S5

Table S1. Raw Counts of transcriptomics of soybean seedlings

Table S2. Differential transcripts of soybean seedlings

Table S3. Gene Ontology (GO) of soybean seedlings transcriptomics

Table S4. Raw reads amplicon sequence variants (ASVs) of soybean seedlings

Table S5. Raw reads differentially expressed genes (DEGs) of soybean seedlings

Table S6. Correlation analysis (Spearman) of BPA treatment and control group by the top 10 (based on the relative abundance and relative expression sort) amplicon sequence variants (ASVs) and differentially expressed genes (DEGs) of soybean seedlings

Table S7. Kyoto Encyclopedia of Genes and Genomes (KEGG) of soybean seedlings transcriptomics
