# Supplementary material for: The impact of Bisphenol A on the endophytic bacterial community and transcriptome of soybean seedlings
Source: iScience. 2025 Mar 13;28(4):112208. doi: 10.1016/j.isci.2025.112208 (PMC12018001; doi:10.1016/j.isci.2025.112208)
Supplement: Document S1. Figures S1–S5 [file mmc1.pdf]

iScience, Volume *28*

Supplemental information

The impact of Bisphenol A on the endophytic bacterial community and transcriptome of soybean seedlings

Ke Wang, Nana Zhong, Manli Yang, Wen Tian, Yaohuan Zhu, Changjiang Huang, Lin Zhao, Xun Liu, Jun Tang, Yuqing Miao, Yuntong Liu, Yu Lei, and Chuansheng Wu

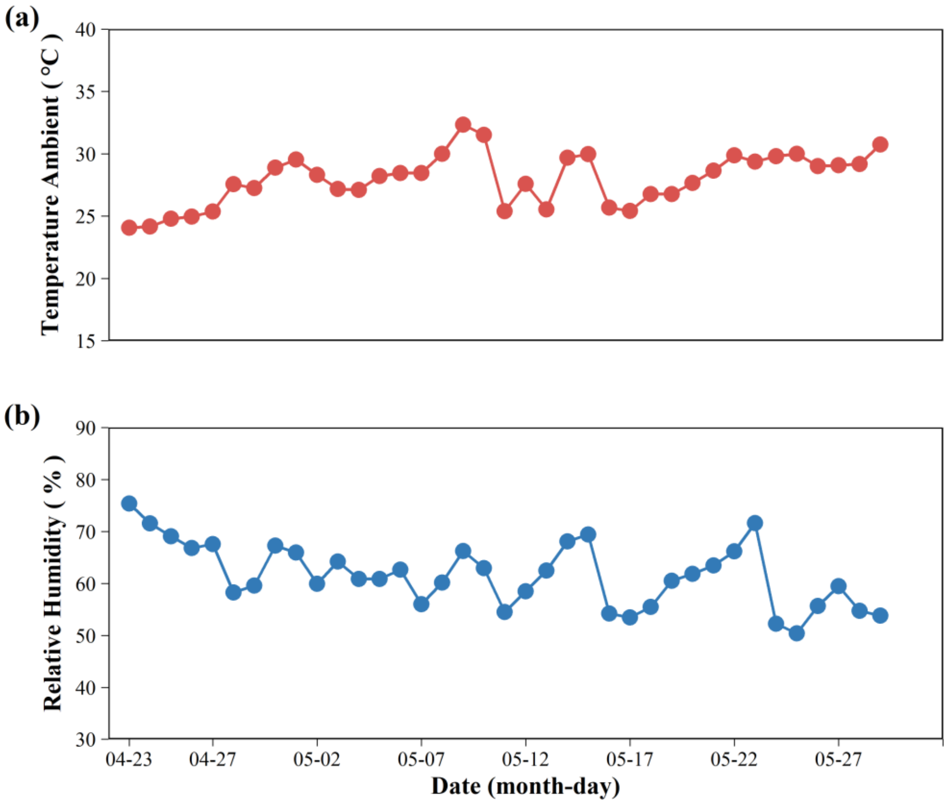

Figure S1. Ambient temperature (a) and relative humidity (b) during pot experiment culture.

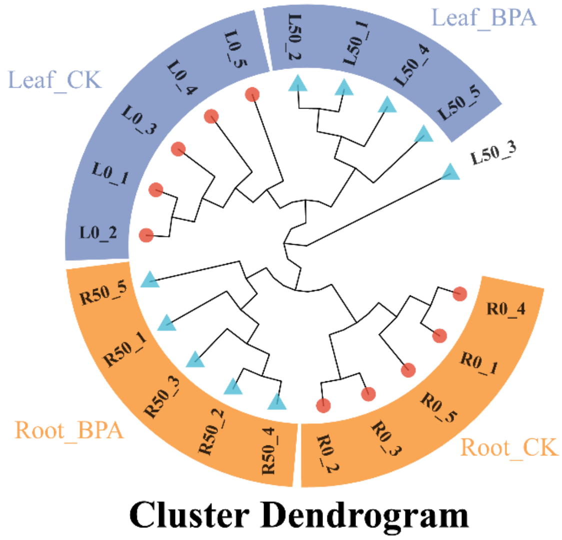

Figure S2. Hierarchical cluster analysis of bacterial endophytes in different samples and treatments. L0 and R0 represent the leaves and roots of soybean seedlings in the control treatment, respectively. L50 and R50 represent the leaves and roots of soybean seedlings in the BPA treatment, respectively.

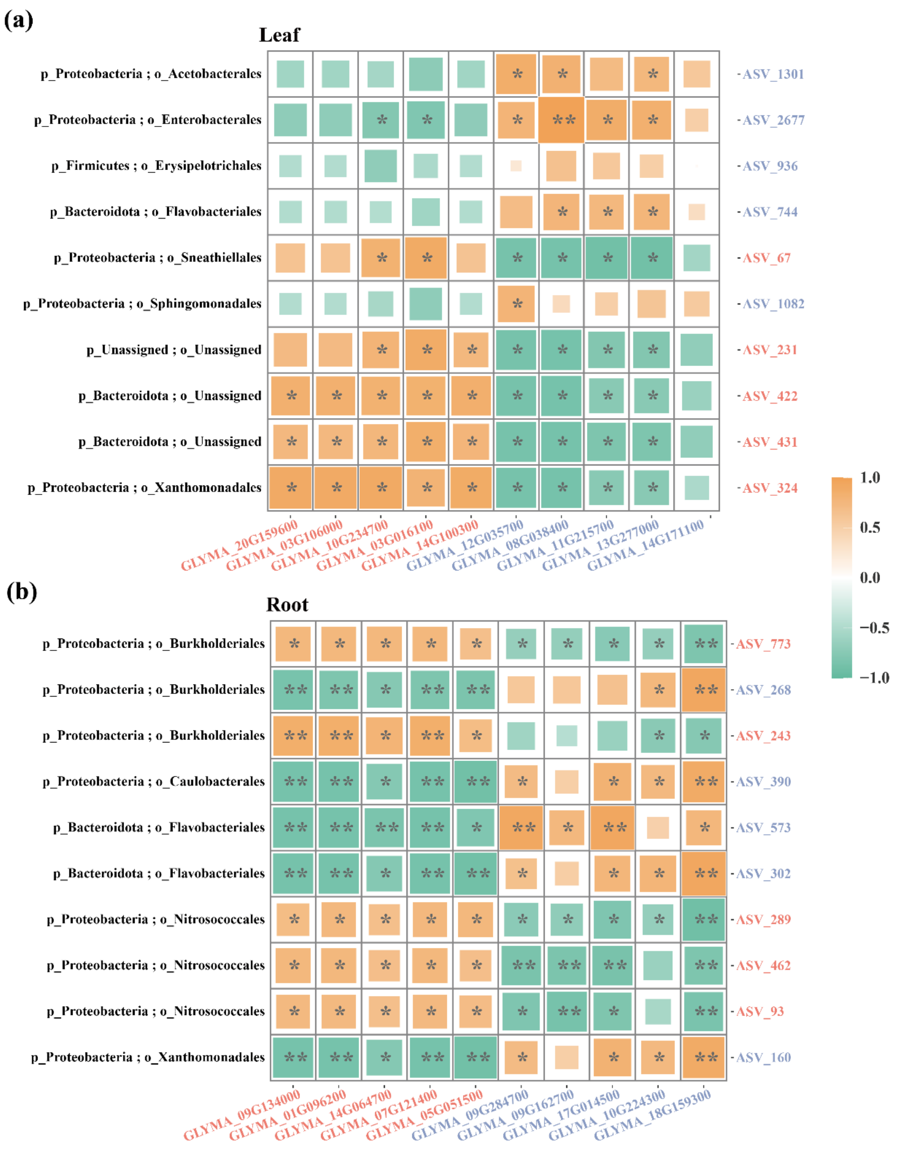

Figure S3. Correlation analysis (Spearman) was performed between the top 5 (sorted according to |log2-fold change| value) upregulated and downregulated differentially expressed genes (DEGs) and amplicon sequence variants (ASVs) in leaves (a) and roots (b) of soybean seedlings between the BPA-treated group and the control group. The DEGs and ASVs were selected based on their |log2-fold change| of ≥ 1 and a p value < 0.5. Each row corresponds to an ASV column to a DEG. The color and asterisks of each cell at the row-column intersection indicate the corresponding correlation coefficient and adjusted p value (Benjamini–Hochberg correction, * $p < 0.05$, ** $p < 0.01$) between the ASV and the DEG. The color from orange to green represents r values from 1 to -1. The red word indicates that the expression of the DEGs or the relative abundance of ASVs is upregulated compared with the control group, while the blue word means downregulation. The black words mark the species annotation information of ASVs (p: phylum, o: order).

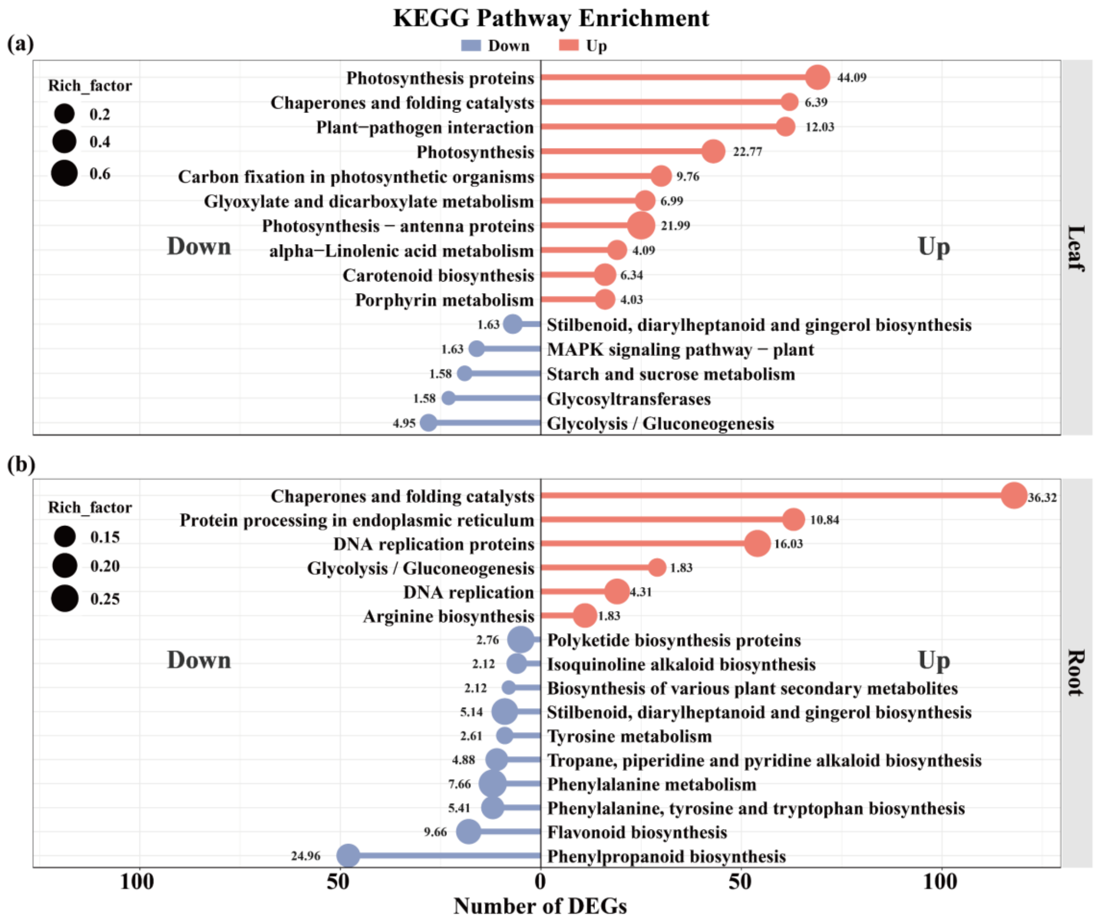

Figure S4. Kyoto Encyclopedia of Genes and Genomes (KEGG) database enrichment analysis showed the significant enrichment of the top 10 pathways (adjusted p value < 0.05, Benjamini–Hochberg correction) of the upregulated and downregulated differentially expressed genes (DEGs) in leaves and roots of soybean seedlings under BPA stress. The value next to each circle represents -log10 (*p* value), where a larger -log10 (*p* value) indicates a more reliable enrichment significance of the DEGs in the pathway. The size of each circle represents the rich factor in the pathways, with larger circles indicating a higher degree of enrichment of the differential genes enriched in the pathways.

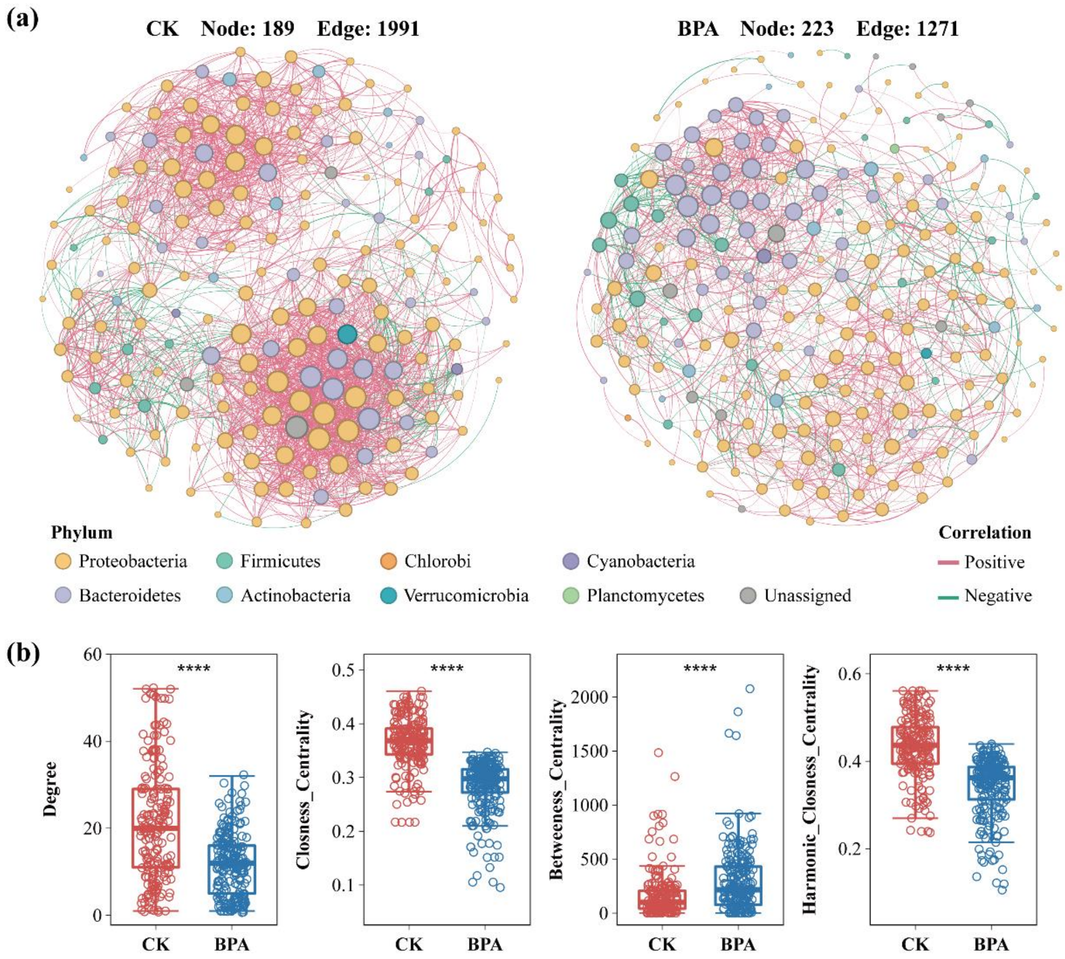

Figure S5. The co-occurrence network of microbial communities (a) and their topological properties (b) in the control group and BPA treatment group based on Spearman's correlation. Nodes indicate individual amplicon sequence variants (ASVs) (threshold is for ASVs with a total relative abundance greater than 0.4% in all samples), while an edge represents a significant correlation between ASVs (Spearman's r > 0.9, FDR-adjusted $p < 0.05$). The size of the node is proportional to the number of connections, and the color of the node represents the phylum to which each ASV belongs. Red lines and green lines represent significant positive and negative connections, respectively, and the width of each edge is proportional to the weight of each node.
